# Perennial Crops Can Compensate for Low Soil Carbon Inputs from Maize in Ley-Arable Systems

**DOI:** 10.3390/plants12010029

**Published:** 2022-12-21

**Authors:** Arne Poyda, Karin S. Levin, Kurt-Jürgen Hülsbergen, Karl Auerswald

**Affiliations:** 1 Institute of Crop Science and Plant Breeding, Grass and Forage Science/Organic Agriculture, Kiel University, Hermann-Rodewald-Str. 9, 24118 Kiel, Germany; 2 Schleswig-Holstein Ministry of Energy, Climate, the Environment and Nature, Mercatorstr. 3, 24106 Kiel, Germany; 3 Chair of Organic Agriculture and Agronomy, Technical University of Munich, Liesel-Beckmann-Str. 2, 85354 Freising, Germany; 4 Aquatic System Biology Unit, Technical University of Munich, Alte Akademie 12, 85354 Freising, Germany

**Keywords:** carbon turnover, carbon isotopes, energy maize, root carbon input, topsoil and subsoil carbon

## Abstract

(1) Background: Soil organic carbon (SOC) in agricultural soils plays a crucial role in mitigating global climate change but also, and maybe more importantly, in soil fertility and thus food security. Therefore, the influence of contrasting cropping systems on SOC not only in the topsoil, but also in the subsoil, needs to be understood. (2) Methods: In this study, we analyzed SOC content and δ^13^C values from a crop rotation experiment for biogas production, established in southern Germany in 2004. We compared two crop rotations, differing in their proportions of maize (0 vs. 50%) and perennial legume–grass leys as main crops (75 vs. 25%). Maize was cultivated with an undersown white clover. Both rotations had an unfertilized variant and a variant that was fertilized with biogas digestate according to the nutrient demand of crops. Sixteen years after the experiment was established, the effects of crop rotation, fertilization, and soil depth on SOC were analyzed. Furthermore, we defined a simple carbon balance model to estimate the dynamics of δ^13^C in soil. Simulations were compared to topsoil data (0–30 cm) from 2009, 2017, and 2020, and to subsoil data (30–60 cm) from 2020. (3) Results: Crop rotation and soil depth had significant effects, but fertilization had no effect on SOC content and δ^13^C. SOC significantly differed between the two crop rotations regarding δ^13^C in both depths but not regarding content. Annual enrichment in C_4_ (maize) carbon was 290, 34, 353, and 70 kg C ha^−1^ per maize year in the topsoil and subsoil of the unfertilized and fertilized treatments, respectively. These amounts corresponded to carbon turnover rates of 0.8, 0.3, 0.9, and 0.5% per maize year. Despite there being 50% maize in the rotation, maize carbon only accounted for 20% of the observed carbon sequestration in the topsoil. Even with pre-defined parameter values, the simple carbon model reproduced observed δ^13^C well. The optimization of model parameters decreased the carbon use efficiency of digestate carbon in the soil, as well as the response of belowground carbon allocation to increased aboveground productivity of maize. (4) Conclusions: Two main findings resulted from this combination of measurement and modelling: (i) the retention of digestate carbon in soil was low and its effect on δ^13^C was negligible, and (ii) soil carbon inputs from maize only responded slightly to increased above-ground productivity. We conclude that SOC stocks in silage maize rotations can be preserved or enhanced if leys with perennial crops are included that compensate for the comparably low maize carbon inputs.

## 1. Introduction

The global amount of organic carbon in soils is approximately 2344 gigatons, which makes it the largest terrestrial organic carbon pool [1]. Arable soils typically contain around 1–3% soil organic carbon (SOC). The SOC content is directly linked to soil organic matter (SOM), which is crucial for the maintenance of many soil functions [2,3]. Carbon turnover of agricultural soils was the focus of several long-term studies and models, describing the processes leading to C input and C output [4,5,6,7,8,9].

Soils under agricultural use are considered to have huge potential as sinks for CO_2_ and for mitigating global climate change [10]. However, ecosystem carbon losses were consistently observed in intensively managed croplands in Europe [11,12,13] in the last decades. In these studies, cropland SOC losses were mainly attributed to the intensification and specialization of conventional cropping systems, with SOC stocks decreasing due to negative SOM reproduction rates. High proportions of silage maize in crop rotations were shown to enhance carbon losses. This was explained by low carbon inputs to the soil from both aboveground plant biomass due to whole-crop harvest [13], and from belowground biomass due to relatively low carbon partitioning to roots [14,15]. Since the latter is comparably high in perennial plant species, their inclusion into rotations of annual crops has the potential to enhance cropland SOC stocks [16]. The perenniality of a crop rotation is therefore considered to be more important with regards to increasing SOC stocks and stabilization efficiency than its crop diversity [17]. Increasing the perenniality of a crop rotation generally means including ley phases with grasses and/or legumes in crop rotations, which were shown to positively affect carbon sequestration [18,19]. In the future, not only management, but also climate change-induced SOC losses, must be offset to maintain or increase SOC stocks [20].

One approach to determine the turnover and residence time of SOC, as affected by individual crops of a crop rotation, is the determination of isotope composition in soil, given that crops differ in their ^13^C to ^12^C ratio, which is usually expressed as δ^13^C. Differences in δ^13^C among crops result from a different fractionation of stable carbon isotopes in the photosynthetic pathway of C_3_ and C_4_ plants [21]. Generally, there is a higher natural abundance of ^13^C in plant tissues of C_4_ plants [22]. Between C_3_ and C_4_ plants, the difference in δ^13^C is approximately 14 ‰ in humid regions with no overlap. Most C_3_ plants have a δ^13^C of −28 to −26‰, while δ^13^C in C_4_ plants ranges from −14 to −12‰ [4,21,23]. The overall fractionation originates from two separate events: different diffusion rates of ^12^C and ^13^C in CO_2_ across the stomata, and the distinct fractionation of ^12^C and ^13^C by the enzyme *Rubisco* [24]. Moreover, carbon fractionation may differ in above- and belowground biomass. It was shown that, in non-woody C_3_ plants, roots are enriched in ^13^C relative to leaves by 1–2‰, whereas C_4_ plant roots had nearly identical or slightly lower δ^13^C values when compared with leaves [25]. These physiologically induced differences between C_3_ and C_4_ plants enable the contributions of C_4_ and C_3_ plant material in mixed crop rotations to SOC to be identified. SOC turnover can therefore be calculated after the input of C_4_ plant material to a soil with SOC predominantly derived from C_3_ plants [5].

Long-term field experiments showed that it is mainly the belowground plant residues that remain in the soil as stabilized SOC [26]. However, there are few controlled experiments studying the carbon turnover of roots due to methodological difficulties in measuring root production and root degradation. Carbon isotopes may, however, provide valuable insights in cases where the input of aboveground material is low. Furthermore, data on the contribution of maize and C_3_ crops to SOC in ley-arable systems is rare, although maize is often grown as ruminant fodder or as a biogas substrate. In Germany, for instance, this applies for 82% of the total maize acreage [27]. This knowledge gap is important because fodder maize can replace leys and vice versa, while a replacement by annual crops would require a change in the farming system and thus is an unlikely option. Furthermore, the amount of residues returned to the soil differs pronouncedly between fodder maize, which leaves only roots and stubbles, and grain maize, where only the kernels are removed. We therefore analyze the contribution of maize roots and stubbles to SOC as compared to the contributions from a ley crop, by comparing the temporal dynamics of soil δ^13^C values in two crop rotations with and without maize. Moreover, SOC and δ^13^C are analyzed at different soil depths as biomass allocation and relative root density are strongly influenced by soil depth, and differences in the allocation to the topsoil and the subsoil between annual crops (maize) and perennial crops (ley) can be expected [28]. Due to lower turnover rates and higher residence times of carbon, the subsoil of many arable fields might still have huge potential as an additional carbon sink [29]. However, most studies only focused on topsoil SOC dynamics.

Both crop rotations of this study contained winter wheat, which, with 26% of the arable land in 2022, is the most important arable field crop in Germany, followed by silage maize (17%). With 5%, grass, legumes, or legume–grass mixtures on arable fields were of minor importance in 2022 compared to the two annual crops [27]. In this study, we use soil and plant measurements from the two crop rotations in a simple carbon balance model to simulate the dynamics of SOC turnover. The main objective of this study is to identify the most important plant-related factors for the turnover and sequestration of SOC in arable crop rotations in the topsoil and subsoil. We hypothesize that (i) higher perenniality in crop rotations increases SOC throughout the soil profile, even when most aboveground material is removed, (ii) the contribution of maize to SOC is lower than its proportion of the rotation, and (iii) SOC dynamics are most strongly affected by plant properties related to belowground carbon allocation. Although our experiment was already running for 16 years, it is unlikely that SOC at the site arrived at an equilibrium, so the quantification of SOC turnover is also required.

## 2. Results

### 2.1. Soil Organic Carbon Content and Isotope Composition

**Figure 1:**
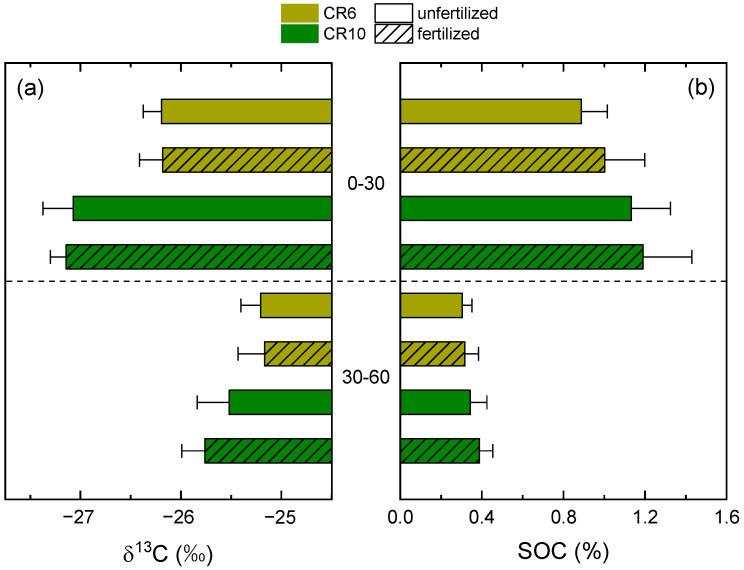
Isotope delta values (**a**) and contents (**b**) of soil organic carbon (SOC) in the 0–30 and 30–60 cm soil layers of CR6 (50% maize) and CR10 (no maize) after 16 years of differences in crop rotation and fertilization. Fertilized treatments received biogas digestate. Soil sampling was conducted in spring 2020. Error bars represent standard deviations.

There were significant differences in SOC content and isotope values between topsoil and subsoil layers (Figure 1, Table 1). Mean δ^13^C was generally less negative in the subsoil and mean values ranged from −27.14‰ in the topsoil of fertilized CR10 to −25.17‰ in the subsoil of fertilized CR6. Mean SOC content was significantly higher in the topsoil. The subsoil in unfertilized CR6 had the lowest SOC content (0.30%), while it was highest in the topsoil in fertilized CR10 (1.19%).

The statistical mixed models explained 88 and 80% of the data variability for δ^13^C and SOC content, respectively. There was a significant interaction of crop rotation with soil depth for both δ^13^C and SOC content (Table 1). However, while δ^13^C was significantly higher in CR6 at both depths, there were no significant differences between CR6 and CR10 regarding SOC content, although it was higher in the topsoil of CR10 for both fertilization treatments. Neither fertilization itself, nor the interaction of fertilization with any other factor, had a significant effect on δ^13^C or SOC content. Consequently, the comparison of fertilized with unfertilized treatments, for which crop rotation and depth were pooled, resulted in non-significant differences for both target variables.

**Figure 2:**
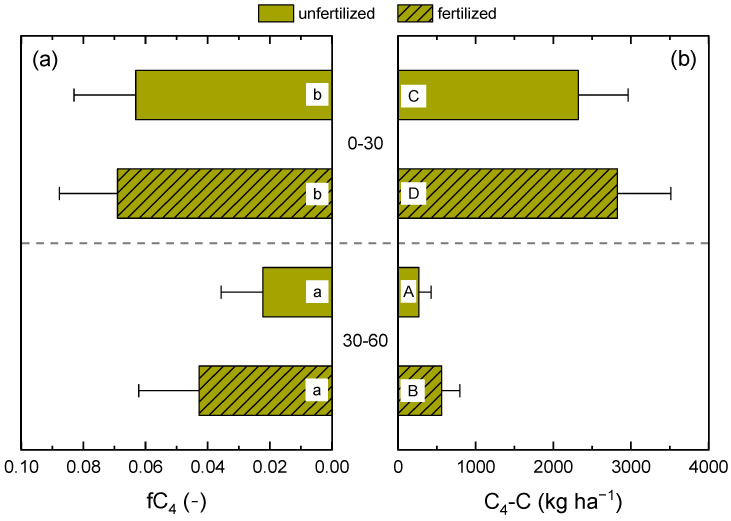
Fractions (**a**) and amounts (**b**) of maize (C_4_) carbon in the soil organic carbon (SOC) stock of the 0–30 and 30–60 cm soil layers of CR6 (50 % maize) compared to CR10 (no maize) after 16 years. Fertilized treatments received biogas digestate. Soil sampling was conducted in spring 2020. Error bars represent standard deviations. Different lowercase and capital letters indicate significant differences between the fractions and amounts of C_4_ carbon, respectively.

Using Equation (2), the amounts and fractions of C_4_ carbon were calculated for the two soil depths and fertilization treatments of CR6 (Figure 2). The amount of C_4_ carbon was significantly higher in the fertilized treatment and in the topsoil, and ranged between 269 kg ha^−1^ in the 30–60 cm layer of the unfertilized treatment and 2830 kg ha^−1^ in the 0–30 cm layer of the fertilized treatment. The proportion of C_4_ carbon in total SOC was 2.2, 4.3, 6.3, and 6.9% in unfertilized and fertilized treatments of the 30–60 and 0–30 cm layers, respectively. However, C_4_ carbon fractions only differed significantly between the topsoil and the subsoil but not between fertilization treatments.

### 2.2. Temporal Development of Carbon Isotope Composition in Topsoil

The statistical mixed effects model for δ^13^C over time explained 74% of data variability. There was a significant interaction between the crop rotation and the year; however, there was no significant effect or interaction for digestate fertilization, as shown by the ANOVA. In autumn 2009, δ^13^C was, on average, 0.30‰ more negative in topsoil SOC of CR10 compared to CR6 (*p* = 0.047), and this difference increased to 0.71‰ in autumn 2017 and 1.09‰ in spring 2020 (both *p* < 0.001) (Table 2). In CR6, no significant differences in topsoil δ^13^C were observed between the years; although, averaged over fertilizer levels, δ^13^C was 0.30‰ less negative in spring 2020 compared to autumn 2009 (*p* = 0.09). In 2017 and 2020, topsoil δ^13^C in CR10 was 0.38 (*p* = 0.019) and 0.49‰ (*p* = 0.002) lower than in 2009, respectively. The difference between 2017 and 2020, however, was not significant (*p* = 0.854).

### 2.3. Modelled δ^13^C Dynamics

#### 2.3.1. Model Performance with Pre-Defined Parameter Values

**Figure 3:**
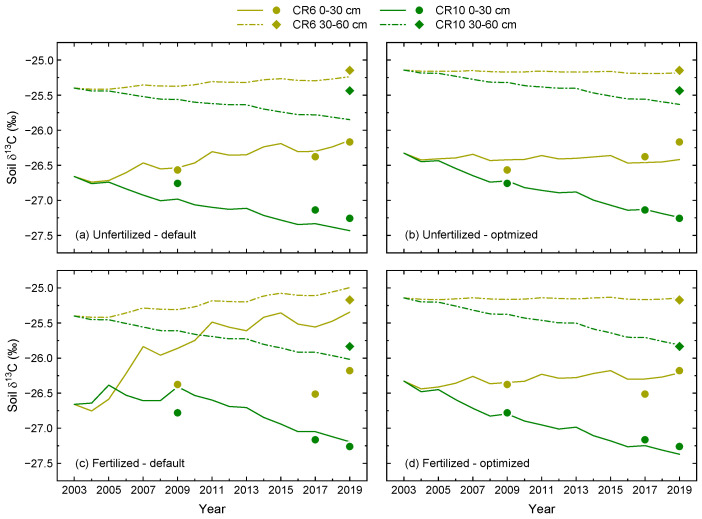
Temporal dynamics of measured (symbols) and modelled δ^13^C (lines) in two different soil layers of CR6 (50% maize) and CR10 (no maize) after the start of the experiment in 2004. (**a**,**c**): Simulations with pre-defined model parameters; (**b**,**d**): Simulations after parameter optimization. Fertilized treatments received biogas digestate. The data points represent averages of four measured δ^13^C values sampled during the dormant period after the respective cropping season.

In the first step, the δ^13^C model (Equation (3)) was run with pre-defined parameter values and 2003 as the first year, the year before the crop rotation experiment was established. Consequently, no treatment effects were expected and homogenous conditions were assumed, as indicated by the same initial δ^13^C values for all treatments at the two soil depths (Figure 3a,c, Table A3).

Simulated δ^13^C increased in CR6 for both fertilization treatments and soil depths. However, this increase was less pronounced in the subsoil compared to the topsoil, and it was greatest in the topsoil of the fertilized treatment where simulations clearly overestimated observed values. Furthermore, fluctuations in δ^13^C due to the rotational cultivation of C_3_ and C_4_ crops became apparent and were also more strongly pronounced in the topsoil. In the unfertilized treatment, the initial model settings reproduced observed δ^13^C in the topsoil of CR6 well. This was also the case for the subsoil layers of both fertilizer treatments, with only small differences between modelled and observed values in 2019.

For CR10, the model showed decreasing δ^13^C values for both fertilization treatments and soil depths (Figure 3a,c). In the topsoil, this decrease in δ^13^C was larger in the unfertilized treatment where observed values were slightly underestimated, while modelled δ^13^C decreased less in the fertilized treatment with a slight overestimation of observed values. In the subsoil, modelled δ^13^C of CR10 decreased more in the fertilized treatment. However, the difference in the observed value at the end of the study period was greater in the unfertilized treatment as the observed δ^13^C was higher.

**Figure 4:**
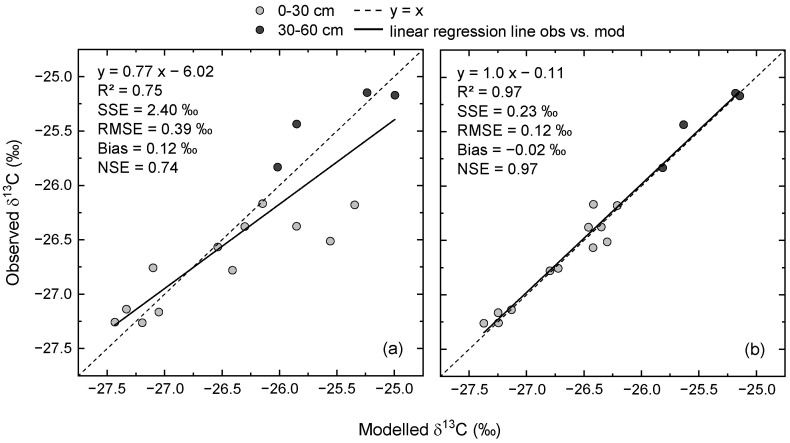
Scatterplots of measured vs. modelled δ^13^C values of soil organic carbon in the topsoil (0–30 cm) and subsoil layer (30–60 cm) using pre-defined parameter values (**a**) and after parameter optimization (**b**). RMSE = root mean squared error, NSE = Nash–Sutcliffe model efficiency. For comparison: the SD of the wheat flour as a blind control was 0.11‰. Each data point is the mean of four plots.

There was a strong relationship between measured and modelled δ^13^C values in SOC, even when based on pre-defined model settings (Figure 4a), as shown by the linear regression (R^2^ = 0.75). The slope of the regression was considerably lower than 1 and the intercept was approximately −6‰, indicating that some observations in the topsoil were overestimated by the model. The RMSE was almost 0.4‰ and the model overestimated the observations on average by 0.12‰, as indicated by the model bias. The NSE of 0.74, which was similar to R^2^, revealed the data scattered around the 1:1 line and that the variability of observations was greater than the variability of errors. Thus, the model with pre-defined parameter values was already a much better predictor than the mean of the measured values.

#### 2.3.2. Parameter Sensitivities and Optimization

The value for SOC δ^13^C at the start of the experiment was unknown and this had greatest impact on model performance, as indicated by the very high sensitivities of δ^13^C SOC_t0_, particularly in the topsoil, but also in the subsoil (Table A3). The sensitivity analysis further indicated that those parameters affecting the amount and depth distribution of belowground C inputs from SM had a major effect on model results (ysi_SM_ > BCA_0–30_SM_ > CUE_SM_ > pf_SM_). In general, the yield-specific index (ysi) was the most sensitive parameter across all crops and it was particularly sensitive for LCG, and also for CG. In contrast, f_HR_ had a negligible effect on model results, which was also the case for all WW-specific parameters.

Optimizing the parameters ysi_SM_, CUE_BD_, δ^13^C SOC_t0_0–30_, and δ^13^C SOC_t0_30–60_ led to the biggest reduction in AIC. Including additional parameters in the optimization process had no further benefits regarding the model results, as indicated by AIC increasing. Optimization resulted in a lower SM ysi and much lower CUE of biogas digestate (Table A4). The value of 0.01 was set as the lower boundary to avoid a mathematically optimized but unfeasible result (i.e., negative CUE). The optimized values of initial δ^13^C were higher compared to the initial values in both the topsoil and subsoil.

Compared to the initial model settings, the optimized model resulted in a better representation of measured δ^13^C dynamics (Figure 3b,d). This was mainly due to less pronounced differences between unfertilized and fertilized treatments. In combination with the increased initial δ^13^C values, the optimized model indicated more stable δ^13^C values over time in CR6 for both fertilizer treatments and soil depths. Except for the topsoil in the fertilized treatment, δ^13^C in CR10 decreased less after optimization. As indicated by the relationship between measured and modelled δ^13^C, parameter optimization resulted in a close-to-perfect model fit (Figure 4b). This is shown by the slope of the linear regression and, in particular, by the NSE of 0.97. Furthermore, the RMSE was reduced to an error level close to the analytical error.

#### 2.3.3. Carbon Allocation

**Figure 5:**
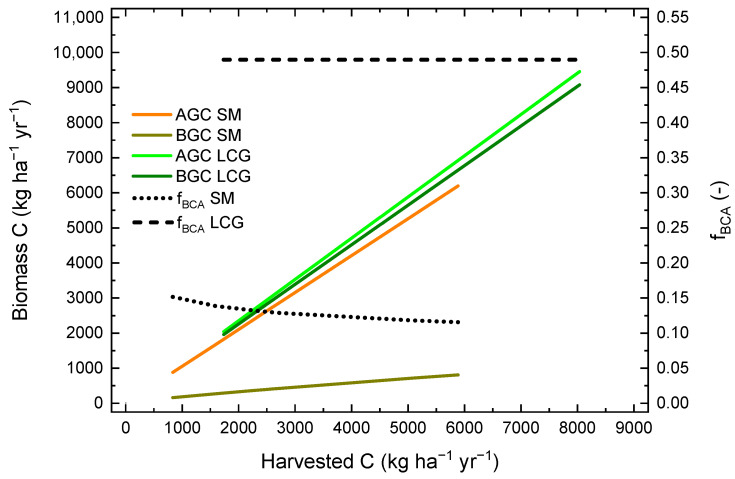
Relationships between modelled carbon in above- (AGC) and belowground biomass in topsoil (0–30 cm) and subsoil (30–60 cm) (BGC), as well as the fraction of carbon that is allocated belowground (f_BCA_) and the carbon in harvested biomass for the two crops silage maize (SM) and lucerne-clover-grass (LCG) over the range of measured crop-specific yields.

Using optimized model parameters, the amounts of carbon in above- and belowground biomass were calculated using Equations (7) and (10) and the constant carbon fraction of 0.47. Furthermore, the fraction of belowground carbon allocation was calculated as the ratio between the carbon in the belowground biomass and in total biomass. The relationships between these three variables and the amount of carbon in harvested biomass are shown in Figure 5 for the crops LCG and SM. In general, aboveground and belowground carbon increased with increasing yield for both crops. However, while aboveground carbon was only slightly higher for LCG compared to SM at the same yield level, belowground carbon was much higher and increased more with yield. At 5000 kg harvested C ha^−1^ yr^−1^, estimated aboveground carbon was 5880 and 5260 kg ha^−1^, and estimated belowground carbon was 5650 and 706 kg ha^−1^ for LCG and SM, respectively. While the fraction of belowground carbon allocation of LCG remained constant at 0.490, it decreased from 0.152 to 0.116 over the range of observed SM yields.

## 3. Discussion

### 3.1. Plant and Soil Effects on SOC Formation

Our results indicate that crop rotation had a significant effect on the content and isotope composition of SOC, while there was no significant fertilization effect. The increase in SOC content between 2009 and 2017 was 500–600 kg ha^−1^ yr^−1^ higher in CR10 (0% maize, 75% leys) compared to CR6 (50% maize, 25% leys), providing strong evidence in support of hypothesis i. In a comparable field experiment on a sandy loam soil in northern Germany [30], stable SOC contents were found for crop rotations including both grass-clover leys and silage maize in one out of three years and with cattle slurry application. Since SOC content decreased under continuous maize and increased under permanent grassland, the authors concluded that the ley proportion needed to be higher than 33% to significantly enhance SOC stocks, as was the case in our study. Although SOC content in 2020 was higher in CR10, particularly in the topsoil, this difference was not significant and the crop rotation effect was much stronger for δ^13^C than for SOC content. This effect was also clearly visible in the subsoil with the difference in δ^13^C between CR6 and CR10 being approximately half as large as the difference in the topsoil.

These findings demonstrate the significant contribution of C_4_ carbon to SOC, even in the subsoil of CR6, where maize was grown in eight of the 16 years since the start of the experiment. Fertilization significantly increased the amounts of C_4_ carbon in the soil, a strong indication that the fertilization with biogas digestate in this system not only increased yields, but also the belowground productivity of maize. This is in contrast to findings by Hirte et al. [31], who observed no difference in belowground carbon inputs of maize, irrespective of aboveground productivity. Interestingly, the fertilized plots also tended to have higher fractions of maize carbon, particularly in the subsoil. Although this difference was not significant, it could be interpreted (with some caution) that digestate fertilization had a larger effect on carbon inputs to the subsoil from maize than from C_3_ crops. Considering the fertilization effect on crop yields, this result is indeed obvious, since fertilization increased maize yields by 112% on average, while winter wheat and clover-grass yields in CR6 only increased by 66 and 18%, respectively. Note that clover-grass in CR6 did not receive digestate directly. Since maize was grown with undersown white clover, which has a shallower rooting system, fertilization likely increased the competitiveness of maize and thereby also led to the formation of a deeper rooting system, resulting in higher carbon inputs to the subsoil.

We found a significant difference in δ^13^C of 1.0 (CR6) and 1.5‰ (CR10) between topsoil and subsoil SOC, which must be due to how the site was cultivated in the years before the start of the experiment, since these differences were even higher than the difference between CR6 and CR10 in the topsoil (0.9‰). In the model approach, the optimization of the initial δ^13^C resulted in a difference of 1.2‰ at the beginning of the experiment. One main reason for this difference is probably related to the Suess effect [32], which was also accounted for in our model. The Suess effect causes recent biomass δ^13^C to be more negative than old biomass δ^13^C under otherwise identical conditions (i.e., identical ^13^C fractionation due to identical photosynthetic pathways and growing conditions) and implies that the δ^13^C of old SOC must be less negative than that of younger SOC. This agrees with our finding that initial δ^13^C in the subsoil was higher, where SOC is older and less influenced by the Suess effect, than in the topsoil. The change in δ^13^C in atmospheric CO_2_ was about 2‰ during the last two centuries, with the rate of change increasing since the 1950s. The difference of 1.2‰ between topsoil and subsoil at the beginning of the experiment is thus not unlikely and very close to the mean difference of 1.1‰ between 0–10 cm (−27.4‰) and 70–100 cm (−26.3‰) in cropland soils without maize and groundwater tables deeper than 200 cm, as determined in the German Agricultural Soil Inventory [33]. In addition to the Suess effect, several other processes potentially contributed to these differences between topsoil and subsoil:

(i) Maize cultivation began in this area in the early 1960s. As changes due to maize carbon would be faster in the topsoil than in the subsoil, this potentially cancelled out a part of the Suess effect. Due to lower carbon turnover in the subsoil, there is most likely a higher fraction of older maize carbon in the subsoil than in the topsoil, explaining the higher initial subsoil δ^13^C. This difference can no longer be measured since the same crops and maize proportions were cultivated over the entire field in the past. (ii) Roots of C_3_ plants are known to have higher δ^13^C values than shoots [25,34]. Given that the subsoil receives carbon mainly from roots while the topsoil also receives a substantial amount of carbon from aboveground crop biomass (particularly in the years before the experiment started when crop residues were left on the field), less negative δ^13^C values in the subsoil are not unlikely. (iii) During the decomposition of organic matter, microbial biomass, which forms only part of SOC, may become enriched by 2‰ compared to SOC, while CO_2_ becomes depleted [35]. However, there is usually negligible fractionation of ^13^C during the transformation of plant residues into SOC [36,37]. Due to these different processes, a difference in initial δ^13^C between topsoil and subsoil is thus not unlikely. It is, however, impossible to disentangle these effects given that the magnitude of the Suess effect, the root effect, and the decomposition effect would be considerably less than 3‰.

The size of topsoil SOC pools increased in all treatments, despite the fact that all aboveground biomass, except for stubbles, was used for biogas production. These observations were recently described for this field trial by Levin et al. [19], who analyzed the change in SOC between 2010 and 2017 for all 10 crop rotations. They found that SOC content increased in 96% of the plots, independent of the type of rotation and the fertilization level. The proportion of clover-grass leys and row crops with undersown clover or clover-grass within a crop rotation had positive and highly significant effects on SOC content. The ley effect was approximately twice as large as the undersowing effect, underlining the generally positive impact of increased perenniality from clover-grass leys on carbon sequestration [18], and given that clover-grass was not grown on that field for several decades before the year 2000. The 0.22% higher SOC content in the 0–30 cm depth of CR10 (75% ley proportion) compared to CR6 (25% ley proportion) found in soil samples from spring 2020 corresponds well with the 0.04% increase in SOC content with every 10% increase in ley proportion identified by Levin et al. [19]. SOC content typically decreases with increasing soil depth in mineral soils due to lower inputs of plant residues, and this was also observed in our study, with significantly lower SOC content in the subsoil. As documented by Schneider et al. [33], climate- and soil-related factors become more important for SOC content than land use at depths below 10 cm.

The amounts of C_4_ carbon found in the soil of CR6 correspond to increments of 34, 290, 70, and 353 kg C_4_-C ha^−1^ per maize cropping year in the subsoil and topsoil of the unfertilized and fertilized treatments, respectively. Despite different pedoclimatic conditions, the values for the topsoil are close to the accumulation rates of 200–300 kg C_4_-C ha^−1^ yr^−1^ in the 0–20 cm depth reported for a long-term maize mono-cropping system in southern China [38]. The resulting carbon turnover, i.e., the increase in the fraction of C_4_ carbon in total SOC per maize year, was 0.8, 0.3, 0.9, and 0.5% in our study. The values for the topsoil fit well with the overall average of 0.8% carbon turnover per year in cropland soils with maize found in the German Agricultural Soil Inventory [33]. In this inventory, however, carbon turnover in the subsoil could not be determined. Irrespective of the fertilization treatment, the enrichment of maize carbon in the topsoil contributed around 20% of the observed overall SOC pool changes of 1409 and 1800 kg ha^−1^ yr^−1^ in the unfertilized and fertilized treatments in CR6. This means that the C_3_ crops contributed 80% of the increase in SOC pool sizes, despite their proportion of the crop rotation only being 50%, which also supports hypothesis ii. However, a C_3_ crop was present on the field during maize years since the white clover cover crop was only mulched and regrew as living mulch in maize. Although the productivity of the white clover was probably low, particularly in the fertilized treatment as described above, there was at least a small input of C_3_ carbon in maize years.

The amounts of 2.3 (unfertilized) and 2.8 Mg ha^−1^ (fertilized) of C_4_ carbon found after sixteen years with eight years of maize cropping in the CR6 plots were remarkably low compared to 2.5–3.2 Mg ha^−1^ found after only two years of conventionally managed maize in a field trial in northern Germany, established after conversion from permanent grassland [15]. This difference can be explained by the much higher productivity of maize crops (shoot biomass of 14–27 Mg dry matter ha^−1^) on the conventionally managed plots in the study by Reinsch et al. [15]. Nevertheless, the huge amounts of SOC these systems lost due to the break-up of the grassland sward could not be offset by the carbon inputs from maize.

Despite the positive effect of digestate fertilization on maize carbon in the soil, this effect was low (+31%) compared to the increase in yield (+112%), and there was no overall fertilization effect on the content or isotopic composition of SOC in the two crop rotations and soil depths. In contrast to these results, the analyses of all plots of the field trial by Levin et al. [19] revealed significantly higher SOC content in fertilized plots. While they found that SOC content was 0.14% higher in fertilized compared to unfertilized plots in autumn 2017, this difference was only 0.06% in the plots sampled for this study in spring 2020. The reason is most likely that the carbon inputs from the two main crops in the crop rotations of this study, legume–grass mixtures and silage maize, had a low sensitivity to fertilization. While the mixtures only showed a small increase in overall productivity, most likely due to a high biological nitrogen fixation of the legume component in the unfertilized treatments [39], maize reacted mainly with increased aboveground productivity, as indicated by the low fraction of belowground carbon allocation.

These results are in line with the frequently observed effect of increasing shoot-to-root ratios with the increasing availability of belowground resources, such as nutrients and water [40,41,42]. Since almost all the aboveground biomass was removed from the field for biogas production in this cropping system, soil carbon inputs from harvest residues were only slightly increased by fertilization. However, in our model, we assumed that the fraction of belowground carbon allocation remained constant with increasing yield, which is mathematically described by the partitioning factor (pf) and the yield-specific index (ysi). The ysi was only reduced for silage maize in the optimization procedure, while a value of 1 was used for the other crops. However, this does not mean that there was no interaction between above- and belowground carbon allocation for these crops; the factor affecting this interaction is not the aboveground productivity directly, but rather the resource availability belowground. The inter-annual yield variability was much higher than the fertilization effect, particularly in the legume–grass mixtures, suggesting that additional important factors had an impact on productivity, and not just nutrient availability.

In addition to the limited effect of fertilization on root carbon inputs in the two crop rotations, the direct effect of digestate carbon on SOC was very low. This was demonstrated by the result that model performance was improved with lower retention of digestate carbon in soil (CUE_BD_). Regarding the mean annual amounts of applied digestate carbon of roughly 1000 and 1100 kg ha^−1^ yr^−1^ in CR6 and CR10, respectively, this result seems to be rather surprising. However, these inputs correspond to 2% of the total SOC stocks and a detectable and significant increase due to these inputs might need even more time than the 16 years of this experiment’s runtime. Digestate fertilization might also potentially increased the decomposition of SOC (positive priming effect), counteracting most of the additional carbon inputs [43]. Very low carbon retention from organic fertilizers (liquid dairy manure) was reported in other studies in mono-cropped cereals [44] and silage maize [30]. The fertilizer-induced carbon retention was at least 20% in cropping systems including perennials in these studies. This value includes both the direct (fertilizer carbon) and indirect (plant carbon) effects. The observed slopes of annual SOC increments in the fertilized crop rotations of this study were 23 (CR10) and 28% (CR6) higher compared to the unfertilized treatments. Assuming that carbon sequestration at the study site will continue for several years until a new equilibrium is reached, the positive effect of biogas digestate application on SOC stocks might be more pronounced in the future.

### 3.2. Modelling SOC Turnover

Using a simple carbon balance model, we could demonstrate that, of the plant-related parameters, those affecting the partitioning between above- and belowground carbon had the biggest impact on model results, thereby confirming hypothesis iii. In general, our model of SOC pool turnover explained the measured δ^13^C values well, even when pre-defined parameter values were used. This was particularly the case for the topsoil in the unfertilized treatments. The effects of both plant carbon on subsoil SOC and digestate carbon on topsoil SOC were overestimated by the preliminary model settings.

Within each of the different plant-related parameter groups (CUE, pf, BCA_0–30_, f_HR_, ysi), the maize-specific parameter was most sensitive regarding its effect on model error. Interestingly, exactly the same reduction in model error was achieved regardless of whether CUE_SM_, pf_SM_, or ysi_SM_ were optimized. In contrast, optimizing the parameter for the allocation of maize carbon between topsoil and subsoil (BCA_0–30_SM_) hardly reduced the model error despite its larger sensitivity compared with CUE_SM_ and pf_SM_. The non-measurable yield-specific silage maize index (ysi_SM_) was selected in the first step of the optimization procedure due to its extremely high sensitivity, in combination with the strong reduction in model error. In addition, the parameters CUE and pf were intentionally kept constant in this model. The partitioning factor for above- and belowground biomass (pf) was taken from literature [45] and is based on the distinct difference between annual and perennial crops. Since all inputs of plant biomass to the soil originated from roots and stubbles, we assumed that the carbon use efficiency of this material did not significantly differ between crops.

The reason for the high sensitivities of maize-specific parameters was that the biggest errors in the preliminary model originated from the large overestimation of δ^13^C in fertilized CR6. Thus, the observed large fertilization effect on maize yields resulted in the estimates of maize carbon inputs to the soil being too high when a constant partitioning to roots was assumed, although this was already much lower compared to the perennial crops. The reduced yield-specific silage maize index resulted in decreasing fractions of belowground carbon allocation with increasing maize yield, and thus a strongly improved model fit for the fertilized CR6. In contrast to Hirte et al. [31], who proposed yield-independent functions for estimating belowground carbon inputs for both maize and wheat, our results suggest crop-specific functions are needed to describe the interactions between yield and belowground carbon allocation.

In the case of maize, our results highlight the minor effect of increased maize yields on soil carbon inputs, as also shown in other studies [46], and confirm the limited contribution maize crops make to the reproduction of soil organic matter and thus carbon sequestration [13]. However, it is very likely that maize, as a valuable crop for forage and biogas production, will remain one of the prevalent crops in Germany, particularly in dairy regions. Therefore, more sustainable maize cropping systems need to be developed. These should involve maize cultivation in rotations with grass-clover leys [15], rather than maize mono-cropping. Our results show that significant carbon sequestration can also be achieved in maize cropping systems if maize is undersown with perennial crops and permanent soil cover is ensured. These systems represent a compromise since undersown clover-grass improves the capacity for carbon sequestration while maize improves biogas yields. To increase the attractiveness of these systems to farmers, research should focus on the effects of different living mulches and undersown crops on maize yields, with a focus on deep-rooting legumes (e.g., lucerne) and herbs (e.g., chicory), as it was shown that these can increase yields of subsequent crops via nutrient transfer from deeper soil layers to the topsoil [47]. The positive effect of such systems will be even larger on sloping sites because undersown leys strongly reduce soil losses due to erosion, and in turn SOC losses. This not only reduces soil losses during the ley phase but also during the subsequent maize phase [48,49].

## 4. Materials and Methods

### 4.1. Experimental Site

All soil samples used in this study originated from the experimental station of the Technical University of Munich (TUM) in Viehhausen, southern Germany. The trial site is on a northeast slope with a gradient of about 9%; 490 m above sea level with annual precipitation of 799 mm yr^−1^ and an annual mean temperature of 9.0 °C (2010–2017). According to US soil taxonomy [50], the soil at the experimental site is categorized as a Hapludalf derived from loess with a silty loam texture and 25% clay down to at least 1 m. Using the World Reference Base [51], the soil is a Haplic Luvisol (Manganiferric, Siltic).

Prior to the crop rotation experiment described in this study, the trial site was used for on-farm research since 1953. At the study site, a long-term crop rotation experiment with 10 different four-year rotations (CR1 to CR10) was running since late 2004. Each crop of every rotation was cultivated each year; the site was hence divided into four blocks. Within each crop rotation, there were eight replicates in each block (four fertilized and four unfertilized). Plot size was set to 6 m width and 12 m length with alleys of 3 m between each row of plots and 9 m between each block. Half of the plots of each rotation were fertilized using only biogas digestate from the anaerobic digestion of biomass for biogas production. The experiment was managed organically.

The aim of the experiment was to quantify the contribution of roots and stubbles to SOC, and in particular the contribution of maize. All aboveground biomass was therefore removed with no returns in the unfertilized treatment. This is an experimental treatment; usually organic matter would be returned to the system, e.g., leys would be mulched. In contrast, a system approach was used in the fertilized treatment; the digestate amount reflected the amount that could be produced with the biomass from the respective rotation and it was divided among the crops according to their estimated nitrogen requirements. Due to the research objective of quantifying the contribution of maize to SOC, the C_4_ proportion in the digestate was low (see below). For more detailed information on the field trial, see Levin et al. [19].

### 4.2. Soil Data

Soil sampling for δ^13^C analyses was conducted in autumn 2009, autumn 2017, and spring 2020 in CR6 (50% silage maize, 25% clover-grass, and 25% winter wheat) and CR10 (0 % silage maize, 75 % lucerne-clover-grass, and 25% winter wheat) since these rotations differed most in terms of the proportion of C_4_ crops and perenniality. In 2009 and 2017, soil samples were taken from 0 to 30 cm soil depth, while in spring 2020 soil was also sampled at 30–60 cm soil depth since changes in isotope composition larger than the measuring error were not expected earlier at this depth. Soil samples were milled using a ball mill with a frequency of 30 s^−1^ for 20 s. For analysis of the ^13^C/^12^C isotope ratio, 10 mg of each soil sample were weighed in 3.3 × 5 mm tin cups. The amount of sample was set according to preliminary experiments to ensure sufficient carbon content for analysis in the isotope ratio mass spectrometer (IRMS). The soil did not contain carbonates that would interfere with isotope measurements of SOC. This was indicated by pH ranging from 6.1 to 6.7 (mean 6.3), a C/N ratio of 9.1 (SD 0.3) in topsoil, and 8.0 (SD 0.4) in the subsoil, by field observations, and also by repeated measurements after HCl fumigation [52] of selected samples of high pH. The carbon isotope composition was determined using an elemental analyzer (NA 1110; Carlo Erba, Milan, Italy) interfaced (ConFlo III; Finnigan MAT, Bremen, Germany) to an IRMS (Delta Plus; Finnigan MAT). Carbon isotopic data are presented as δ^13^C, with

δ^13^C = R_sample_/R_standard_ − 1, (1)

where R is the ^13^C/^12^C ratio and R_standard_ denotes the Vienna Pee Dee Belemnite standard. Each sample was measured against a laboratory working standard CO_2_ gas, which was previously calibrated against an IAEA secondary standard (IAEA-CH6). After every tenth sample, wheat flour with a similar amount of carbon to the weighed sample material (differing for top and subsoil samples) was measured as a blind control. The wheat flour was calibrated against the international standard (IAEA-CH6). Accuracy, quantified as standard deviation (SD) of wheat flour replicates, was 0.11‰.

### 4.3. Plant Data

In CR10, the lucerne-clover-grass was sown directly after the wheat harvest in autumn to avoid long fallow periods. In CR6, the clover-grass was usually sown in spring due to the late harvest of silage maize. After the winter wheat harvest, white clover or a cereal–white clover mixture was sown as a cover crop, which was mulched before maize sowing. Thus, the white clover regrew as living mulch and was regularly mulched during the two maize years. The aboveground dry matter productivity of the clover crop was 2.4 and 3.0 Mg ha^−1^ yr^−1^ in the unfertilized and fertilized plots, respectively. In contrast to the aboveground biomass of the main crops, the white clover biomass remained on the field. Due to the different sowing times, lucerne-clover-grass was cut 4–5 times per year, while clover-grass was only cut 2–3 times per year. All harvest yields were measured using a plot harvester. The wheat straw biomass that was removed from the field was measured manually to obtain the total aboveground biomass and the straw/grain ratios (Table 1).

The δ^13^C of aboveground biomass (δ^13^C_AGB_) was measured for silage maize (−13.2‰, n = 6), winter wheat (−26.9‰, n = 6), and legume–grass mixtures (−29.7‰, n = 127) using the IRMS (Table 1). No δ^13^C measurements were available for cover crop and root biomass. Thus, the mean δ^13^C of C_3_ biomass (winter wheat and legume–grass mixtures) was used for cover crops. The δ^13^C of belowground biomass (δ^13^C_BGB_) was estimated using δ^13^C_AGB_ + 1‰ for C_3_ crops and δ^13^C_AGB_ for maize, in accordance with Badeck et al. [53]. To be consistent with the SOC δ^13^C data, which was only measured for block 3, yield data from block 3 were used as input data for the time series analysis of crop rotation effects on SOC (Table 3). For those years where yield data were not available, mean yields from the period 2009 to 2017 were used.

The fraction of maize-derived carbon that accumulated in SOC in CR6 since the beginning of the experiment (fC_4_) was derived from δ^13^C in the soil sample (δ^13^C_SOC_) according to Balesdent et al. [54]:

fC_4_ = (δ^13^C_SOC_ − δ^13^C_ref_)/(δ^13^C_C4_ − δ^13^C_ref_), (2)

where δ^13^C_ref_ = mean δ^13^C in soil samples from CR10 in the same year and fertilizer treatment, and δ^13^C_C4_ = the δ^13^C of maize biomass.

### 4.4. Digestate Data

The biogas digestate was produced by a local organic farmer from a mix of different feedstocks. Annual amounts of digestate, as given by Levin et al. [19], were assumed to repeat regularly in each four-year crop rotation (Table A1). Biogas substrate composition for the period 2009–2017 was, on average, 61% silage from (lucerne-) clover-grass and grassland biomass, 30% solid cattle manure, 5% silage maize, and the remainder cereal grains. From 2010 on, the composition gradually changed to larger fractions of C_3_ plant material and cattle manure, and only the liquid phase of digestate was used in this trial. Dry matter content in the liquid phase was 7.9%, of which, 39% was carbon. The δ^13^C of biogas digestate (δ^13^C_BD_) was calculated according to the fractions of C_3_ and C_4_ material used as substrates, where cattle manure was treated as C_3_ material due to the cattle’s diet (Table A1). For 2004–2009, the δ^13^C_BD_ of 2010 was used, while the mean δ^13^C_BD_ for the period 2012–2017 was used for 2018 and 2019 since almost identical fractions of maize substrate were used from 2012 onward.

### 4.5. Statistical Analyses of Soil Carbon Data

Data evaluation using the software package R [55] started with the definition of appropriate statistical mixed models [56,57]. In order to analyze changes in δ^13^C in topsoil data between 2009, 2017, and 2020, the model included crop rotation, fertilization and year, and their interaction terms as fixed factors. The rows and plots were defined as random factors. The effects of different soil depths on SOC, δ^13^C, and fC_4_ were evaluated with a second model using data from 2020. Thus, instead of the year, the soil depth was included as a fixed factor and the block effect was defined as an additional random factor. Furthermore, the model took into account the correlation of measurements from the two depths of the same plot. The residuals of the δ^13^C and fC_4_ models were assumed to be normally distributed and homoscedastic, while the residuals of the SOC model were assumed to be normally distributed and heteroscedastic due to the different soil depths. These assumptions were based on a graphical analysis of the residuals. In the next step, a pseudo R^2^ was calculated [58], indicating the fraction of variability of the target variable that could be explained by the mixed model, and an analysis of variance (ANOVA) was conducted. In order to compare the different levels of the influence factors, multiple contrast tests [59] were performed. If a factor of interest had no significant interactions with the remaining factors, then the levels of these remaining factors were pooled.

### 4.6. Modelling the Temporal Development of δ^13^C in the Soil

#### 4.6.1. Model Structure

We used a model to predict the temporal development of δ^13^C in soil, which included the effects of the crops cultivated and fertilization. The model took into account the ongoing change in δ^13^C values in all primary biomass and subsequent pools due to changing δ^13^C in atmospheric CO_2_, also referred to as the Suess effect [32,60]. When using the atmospheric CO_2_ data measured at Mauna Loa within the Scripps CO_2_ program, mean annual δ^13^C linearly decreased in atmospheric CO_2_ between 1994 and 2020 by 0.0281‰ per year (R^2^ = 0.988, n = 27). Over the entire study period between the first growing season in 2005 and the last growing season in 2019, the total Suess effect amounted to (14 × 0.0281 =) 0.39‰. Our model used a mass balance equation, which included the SOC stock of the previous time step (SOC_t−1_) of a certain soil layer, the annual C inputs to this layer (C_in_), and the δ^13^C values of SOC and input components. We included the aboveground biomass (stubbles and cover crops), the belowground biomass, and the application of biogas digestate (all inputs in kg ha^−1^ yr^−1^), when calculating the annual carbon input.

δ^13^C (SOC_t_) = (SOC_t−1_ * δ^13^C (SOC_t−1_) + C_in_AGB_ * δ^13^C_AGB_ + C_in_BGB_ * δ^13^C_BGB_ + C_in_BD_ * δ^13^C_BD_)/(SOC_t−1_ + C_in_AGB_ + C_in_BGB_ + C_in_BD_) (3)

The initial SOC stocks of different soil layers (SOC_t0_L_, kg ha^−1^) were calculated from dry bulk density (d_B_L_, kg m^−3^), the C content in the respective soil layer (C_soil_L_, %), and the depth of the soil layer (D_L_ = 30 cm).

SOC_t0_L_ = d_B_L_ * C_soil_L_ * D_L_ (4)

Since the SOC stocks at the study site were not in an equilibrium state but were considerably affected by crop rotations as documented by Levin et al. [19], the changes in topsoil SOC between 2009 and 2017 were included in the model (Table A2). Therefore, multiple linear regression was used to obtain the change in SOC stock (ΔSOC Δt^−1^, kg yr^−1^) for every crop rotation and fertilization treatment, using the same intercept due to the homogeneous management of the field before the start of the experiment. For the 30–60 cm depth, the lowest observed SOC stock (unfertilized CR6) was assumed to be constant and the differences to the other treatments were evenly attributed as a linear increase over the experimental period. The SOC stock for a specific soil depth at time t (SOC_t_L_, kg ha^−1^) was then calculated using linear regression:

SOC_t_L_ = SOC_t0_L_ + (ΔSOC/Δt)_L_ * t (5)

The C input from the aboveground biomass of a specific crop (kg ha^−1^) to SOC was calculated as follows:

C_in_AGB_ = AGB * f_HR_ * C_plant_ * CUE_AGB_, (6)

where f_HR_ = a crop-specific factor for the fraction of aboveground harvest residues of the total aboveground biomass (-); C_plant_ = the carbon fraction in dry crop biomass using a constant factor of 0.47, as recommended by the IPCC for herbaceous biomass from grassland and cropland [61]; CUE_AGB_ = the efficiency with which carbon from aboveground biomass is converted to microbial or humified organic matter, and thus retained in the soil (-). The distribution of C_in_AGB_ in the soil profile was restricted to the ploughing depth, which was similar to the sampling depth of 0–30 cm.

The annual aboveground biomass was estimated using f_HR_ and yields (kg ha^−1^ yr^−1^) for crops harvested for whole crop silage (clover-grass, lucerne-clover-grass, and silage maize) and the straw/grain ratio (-) for winter wheat. For years with cover crop and clover living mulch in CR6, the aboveground biomass of the cover crop was added to the aboveground biomass of the main crop (where f_HR_CC_ = 1).

AGB_SM,CG,LCG,CC_ = Yield/(1 − f_HR_) (7)

AGB_WW_ = (Yield + Yield * (Straw/Grain))/(1 − f_HR_) (8)

To distribute the belowground C input among different soil layers (C_in_BGB_L_, kg ha^−1^ yr^−1^), the belowground biomass was multiplied with the constant carbon fraction of 0.47 (C_plant_), the carbon use efficiency of belowground biomass (CUE_BGB_, -), and a factor that allocated a certain proportion of the belowground carbon to the specific layer (BCA_L_, -):

C_in_BGB_L_ = BGB * C_plant_ * CUE_BGB_ * BCA_L_ (9)

The belowground biomass was calculated by multiplying aboveground biomass with a partitioning factor (pf, -), equivalent to the ratio between belowground and aboveground biomass. In addition, a yield-specific index (ysi) was introduced to take potential interactions between the amount of aboveground biomass and pf into account:

BGB = (pf * AGB)^ysi.^ (10)

The carbon input to a soil layer from the application of biogas digestate (C_in_BD_L_, kg ha^−1^ yr^−1^) was calculated as follows:

C_in_BD_L_ = A_BD_ * DM_BD_ * D_BD_ * C_BD_ * CUE_BD_ * f_BD_L_, (11)

with A_BD_, DM_BD_, D_BD_, C_BD_, CUE_BD_, and f_BD_L_, the amount (m^3^ ha^−1^ yr^−1^), dry matter fraction (0.079), density (assumed to be 1000 kg m^−3^), carbon fraction of dry matter (0.39), carbon use efficiency (-), and the fraction of biogas digestate allocated to the soil layer (-), respectively.

Modelling was performed for the period 2004–2019 using annual increments, thus carbon inputs were calculated based on the crop yields (Table 3) and digestate (Table A1) amounts for each specific year. Mean yields for the period from 2009 to 2017 were used for the years outside this period. In addition to the procedure described above, the calculation of the δ^13^C of carbon inputs accounted for the Suess effect for inputs of both plant material and biogas digestate. The mean δ^13^C value from the unfertilized plots of both crop rotations (−26.66‰) measured in 2009 was used as the initial δ^13^C of topsoil SOC, as no earlier data were available. For the 30–60 cm layer, the overall mean of measurements from 2020 (−25.40‰) was used as the initial value. These initial values were adjusted during the optimization procedure, as described below.

#### 4.6.2. Model Performance and Parameter Optimization

Modelled δ^13^C was compared to measurements in autumn 2009 and 2017, as well as spring 2020. As spring measurements reflect soil conditions affected by crops grown in the previous year, the spring 2020 data were compared to the model output for 2019. The ability of the model to reproduce the observations was evaluated using different indicators. Firstly, to determine whether the model tended to over- or underestimate the observations, the mean model bias was calculated as follows:

Bias = (1/n) * ∑ (δ^13^C^mod^(i,L) − δ^13^C^obs^(i,L)), (12)

where n = the number of observations; δ^13^C^mod^(i,L) and δ^13^C^obs^(i,L) = the modelled and observed δ^13^C values for time i and soil layer L, respectively. The sum of squared errors (SSE) is given by:

SSE = ∑ (δ^13^C^mod^(i,L) − δ^13^C^obs^(i,L))^2.^ (13)

The root mean squared error (RMSE) can then be interpreted as the mean deviation from the perfect fit of the modelled vs. measured values.

RMSE = √ ((1/n) * ∑ (δ^13^C^mod^(i,L) − δ^13^C^obs^(i,L))^2^) (14)

Finally, the accuracy of model prediction was evaluated using the Nash–Sutcliffe model efficiency (NSE) coefficient:

NSE = 1 − ((∑ (δ^13^C^mod^(i,L) − δ^13^C^obs^(i,L))^2^)/(∑ (δ^13^C^obs^(i,L) − mean(δ^13^C^obs^(L)))^2^)), (15)

where mean(δ^13^C^obs^(L)) is the mean observed δ^13^C of SOC in soil layer L. The NSE compares the error variance (the numerator) with the variance of observations (the denominator) and has a maximum of 1, equivalent to a perfect model prediction. If 0 < NSE ≤ 1, model predictions are more accurate than the mean of the measured data, while NSE < 0 indicates that the mean of the measured data predicts the δ^13^C of SOC better than the model.

The parameter optimization procedure used a stepwise approach. To identify those parameters with the highest impacts on model results, a sensitivity analysis was performed as the first step. Therefore, initial parameter values (Table A3) were altered by 10% and the corresponding relative change in SSE was used as parameter sensitivity. In the next step, the absolute change in SSE after optimizing the individual parameters was analyzed. The optimization procedure was conducted using the “Solver” analytical tool in the Excel program. The tool iteratively varies the parameter values within pre-defined thresholds to identify the optimal parameter combination, resulting in the lowest value of the target variable (SSE). Those parameters with the highest interaction between sensitivity and absolute model improvement were selected for optimization, aiming for a parameter combination that minimized the Akaike information criterion (AIC). The AIC accounts for the number of observations (n), the number of optimized parameters (K), and the resulting SSE after optimization of the respective parameters in the previous step [62].

## 5. Conclusions

This study demonstrated that the crops cultivated on arable soils have a significant impact on SOC, not only in the ploughed topsoil, but also in the subsoil. The carbon turnover in the layer beneath the plough layer was still 40–50% of that in the topsoil. While the type of crop rotation strongly affected SOC, fertilization with biogas digestate did not. The annual contribution of maize to SOC sequestration (20%) was much lower than its proportion of the crop rotation (50%). Under the conditions of this study, the following conclusions can be drawn: (i) SOC in the topsoil and subsoil differs strongly in its isotopic composition, (ii) the effect of digestate carbon on δ^13^C is negligible, and (iii) maize carbon inputs to soil only respond slightly to increased aboveground productivity. Given these findings, we conclude that SOC stocks in silage maize cropping systems can only be increased or maintained at higher levels when legume-grass leys are included in the rotations. This is not only required to mitigate CO_2_ emissions from agricultural soils, but also to increase the resilience of cropping systems against the impacts of erosion and climate change, and to maintain soil fertility and thereby food security.

## Figures and Tables

**Table 1:** Analysis of variance (ANOVA) and comparisons of means for content and isotope delta values of soil organic carbon (SOC) in soil samples taken from two different crop rotations, soil depths and fertilizer treatments after 16 years of differences in crop rotation and fertilization. Effects or differences were considered as non-significant (n.s.) for *p* ≥ 0.05, and level of significance is indicated as follows: 0.05 > *p* ≥ 0.01: *, *p* < 0.001: ***.

	SOC			δ^13^C		
ANOVA	*p* Value			*p* Value		
Crop rotation	0.016		*	<0.001		***
Fertilization	0.184		n.s.	0.402		n.s.
Depth	<0.001		***	<0.001		***
Crop rotation:Fertilization	0.428		n.s.	0.242		n.s.
Crop rotation:Depth	0.011		*	<0.001		***
Fertilization:Depth	0.366		n.s.	0.458		n.s.
Crop rotation:Fertilization:Depth	0.471		n.s.	0.306		n.s.
**Comparisons**	**Difference**			**Difference**		
CR6 vs. CR10 (0–30)	−0.22	1	n.s.	0.92	<0.001	***
CR6 vs. CR10 (30–60)	−0.06	1	n.s.	0.45	<0.001	***
0–30 vs. 30–60 (CR6)	0.64	<0.001	***	−1.00	<0.001	***
0–30 vs. 30–60 (CR10)	0.80	<0.001	***	−1.47	<0.001	***
Unfertilized vs. fertilized	−0.06	0.143	n.s.	0.07	0.402	n.s.

**Table 2:** Isotope delta values of soil organic carbon in the 0–30 cm layer of CR6 (50% maize) and CR10 (no maize) in three different years after the start of the experiment in 2004. Fertilized treatments received biogas digestate. Different uppercase letters represent significant differences between crop rotations in a specific year and different lowercase letters represent significant differences between years in a specific crop rotation, both at a significance level of *p* < 0.05. Due to no significant effect or interaction of fertilization, data were pooled over fertilizer treatments prior to comparisons of means.

Year	δ^13^C in Topsoil (‰)					
	CR6 Unfertilized	CR6 Fertilized		CR10 Unfertilized	CR10 Fertilized	
2009	−26.57	−26.38	Ba	−26.76	−26.78	Ab
2017	−26.38	−26.51	Ba	−27.14	−27.17	Aa
2020	−26.17	−26.18	Ba	−27.26	−27.27	Aa

**Table 3:** Mean annual dry matter yields of crop rotations CR6 and CR10 in unfertilized plots and plots fertilized with biogas digestate (n = 4). WW: winter wheat (grain only), SM: silage maize, CG: clover-grass mixture, LCG: lucerne-clover-grass mixture, CC: cover crop (not harvested), and S/G: straw/grain ratio.

Year	CR6			CR10		
	Crop	Unfertilized	Fertilized	Crop	Unfertilized	Fertilized
		t ha^−1^ yr^−1^			t ha^−1^ yr^−1^	
2009	WW	4.2	6.7	WW	4.1	6.6
2010	SM	3.5	6.1	LCG	10.2	12.1
2011	SM	5.6	11.4	LCG	4.5	6.8
2012	CG	3.9	4.7	LCG	3.9	7.8
2013	WW	3.5	7.6	WW	3.0	7.3
2014	SM	5.8	13.1	LCG	15.1	18.3
2015	SM	1.9	5.0	LCG	10.3	11.9
2016	CG	9.7	11.4	LCG	10.6	14.5
2017	WW	4.7	6.1	WW	2.8	4.9
Mean	CG	6.8	8.0	LCG	9.1	11.9
	WW	4.1	6.8	WW	3.3	6.2
	SM	4.2	8.9			
	CC	2.4	3.0			
S/G	WW	1.13	1.05	WW	1.27	1.04

## Data Availability

The data presented in this study are available on request from the corresponding author.

## Appendix A

**Table A1:** Annual amounts of biogas digestate applied to fertilized plots of crop rotations CR6 (50 % maize) and CR10 (no maize), as well as δ^13^C values of the applied biogas digestate (δ^13^C_BD_) calculated from the fractions of C_3_ and C_4_ biomass used in the biogas plant and accounting for the terrestrial Suess effect [32].

Year	Biogas Digestate (m^3^ ha^−1^ yr^−1^)		δ^13^C_BD_ (‰)
	CR6	CR10	
2004	0	50	−20.58
2005	43	77	−20.61
2006	45	0	−20.64
2007	44	20	−20.66
2008	0	50	−20.69
2009	43	77	−20.72
2010	45	0	−25.13
2011	44	20	−26.97
2012	0	50	−27.90
2013	43	77	−28.08
2014	45	0	−27.81
2015	44	20	−28.29
2016	0	50	−28.01
2017	43	77	−27.74
2018	45	0	−28.07
2019	44	20	−28.10

**Table A2:** Topsoil- and subsoil-specific parameters with constant values used in the model.

Parameter	Unit	Application	Value
Topsoil			
SOC_t0_	kg ha^−1^	General	35,345
d_B_	kg m^−3^	General	1400
ΔSOC Δt^−1^	kg yr^−1^	CR6 unfertilized	1409
ΔSOC Δt^−1^	kg yr^−1^	CR6 fertilized	1800
ΔSOC Δt^−1^	kg yr^−1^	CR10 unfertilized	1961
ΔSOC Δt^−1^	kg yr^−1^	CR10 fertilized	2415
f_HR_CC_	-	General (CC only present in CR6)	1.00
Subsoil			
SOC_t0_	kg ha^−1^	General	14,124
d_B_	kg m^−3^	General	1450
ΔSOC Δt^−1^	kg yr^−1^	CR6 unfertilized	0
ΔSOC Δt^−1^	kg yr^−1^	CR6 fertilized	97
ΔSOC Δt^−1^	kg yr^−1^	CR10 unfertilized	180
ΔSOC Δt^−1^	kg yr^−1^	CR10 fertilized	348

**Table A3:** Initial parameter values and sensitivities of model parameters used in the model. Sensitivity was quantified as being a 10% variation in the parameter values when keeping the other parameters constant and calculating the percentage change in the sum of squared errors (SSE) compared to the SSE calculated with the initial value. References are given for those parameter values that were obtained from literature.

Parameter	Initial Value	Sensitivity (%)	Reference
δ^13^C SOC_t0_0–30_ (‰)	−26.66	1708.5	
δ^13^C SOC_t0_30–60_ (‰)	−25.40	763.6	
f_BD_0–30_	1.00	5.3	
CUE_BD_	0.60	5.5	
CUE_LCG_	0.40	3.6	
CUE_CG_	0.40	5.8	
CUE_WW_	0.40	0.0	
CUE_SM_	0.40	22.0	
CUE_CC_	0.50	2.5	
pf_LCG_	0.96	3.0	[45]
pf_CG_	0.96	4.7	[45]
pf_WW_	0.47	0.1	[45]
pf_SM_	0.47	19.6	[45]
pf_CC_	0.96	0.9	[45]
BCA_0–30_LCG_	0.85	0.3	
BCA_0–30_CG_	0.95	5.2	
BCA_0–30_WW_	0.90	0.2	
BCA_0–30_SM_	0.90	25.2	
BCA_0–30_CC_	0.90	0.5	
f_HR_LCG_	0.15	1.0	[14]
f_HR_CG_	0.15	2.2	[14]
f_HR_WW_	0.05	0.1	[14]
f_HR_SM_	0.05	3.2	[14]
ysi_LCG_	1	121.7	[63]
ysi_CG_	1	45.1	[63]
ysi_WW_	1	2.7	[63]
ysi_SM_	1	436.9	[63]
ysi_CC_	1	9.8	[63]

**Table A4:** Initial and final values of optimized parameters, the sum of squared errors (SSE), and Akaike’s information criterion (AIC). Parameters are listed in order according to the optimization process.

Parameter/Indicator	Initial Value	Final Value
ysi_SM_	1	0.84
CUE_BD_	0.60	0.01
δ^13^C SOC_t0_0–30_ (‰)	−26.66	−26.33
δ^13^C SOC_t0_30–60_ (‰)	−25.40	−25.14
SSE	2.40	0.23
AIC	−27.07	−46.02
